# Immunomodulatory Effect of Chinese Herbal Medicine Formula Sheng-Fei-Yu-Chuan-Tang in Lipopolysaccharide-Induced Acute Lung Injury Mice

**DOI:** 10.1155/2013/976342

**Published:** 2013-08-13

**Authors:** Chia-Hung Lin, Ching-Hua Yeh, Li-Jen Lin, Shulhn-Der Wang, Jen-Shu Wang, Shung-Te Kao

**Affiliations:** ^1^Graduate Institute of Chinese Medical Science, China Medical University, Taichung 404, Taiwan; ^2^Department of Medicinal Botanicals and Health Care, Da-Yeh University, Changhua 515, Taiwan; ^3^School of Chinese Medicine, College of Chinese Medicine, China Medical University, Taichung 404, Taiwan; ^4^School of Post-Baccalaureate Chinese Medicine, College of Chinese Medicine, China Medical University, Taichung 404, Taiwan; ^5^Institute of Medical Science, Tzu Chi University, Hualien 427, Taiwan; ^6^Taichung Tzu Chi General Hospital, Taichung 970, Taiwan; ^7^Department of Chinese Medicine, China Medical University Hospital, Taichung 404, Taiwan

## Abstract

Traditional Chinese medicine formula Sheng-Fei-Yu-Chuan-Tang (SFYCT), consisting of 13 medicinal plants, was used to treat patients with lung diseases. This study investigated the immunoregulatory effect of SFYCT on intratracheal lipopolysaccharides- (LPS-) challenged acute lung injury (ALI) mice. SFYCT attenuated pulmonary edema, macrophages, and neutrophils infiltration in the airways. SFYCT decreased inflammatory cytokines, including tumor necrosis factor-**α** (TNF**α**), interleukin-1**β**, and interleukin-6 and inhibited nitric oxide (NO) production but increased anti-inflammatory cytokines, interleukin-4, and interleukin-10, in the bronchoalveolar lavage fluid of LPS-challenged mice. TNF**α** and monocyte chemotactic protein-1 mRNA expression in the lung of LPS-challenged mice as well as LPS-stimulated lung epithelial cell and macrophage were decreased by SFYCT treatment. SFYCT treatment also decreased the inducible nitric oxide synthase expression and phosphorylation of nuclear factor-**κ**B (NF-**κ**B) in the lung of mice and macrophage with LPS stimulation. SFYCT treatment dose dependently decreased the LPS-induced NO and reactive oxygen species generation in LPS-stimulated macrophage. In conclusion, SFYCT attenuated lung inflammation during LPS-induced ALI through decreasing inflammatory cytokines production while increasing anti-inflammatory cytokines production. The immunoregulatory effect of SFYCT is related to inhibiting NF-**κ**B phosphorylation.

## 1. Introduction

Acute lung injury (ALI) is characterized by overwhelming lung inflammation with recruitment and activation of neutrophils and macrophages [1]. ALI is associated with the development of interconnected inflammatory cascades, with proinflammatory cytokines playing a central role in the initiation and propagation of the inflammatory response leading to lung injury. Bacterial superinfection of the lung is a common complication of ALI [2, 3]. Several animal models have been developed to study the pathophysiologic mechanisms involved in ALI [4, 5]. In particular, the intratracheal administration of lipopolysaccharide (LPS) has gained wide acceptance as a clinically relevant model of ALI [4, 6, 7]. The inhalation of LPS results in acute, neutrophilic inflammation of the distal air spaces of the lungs. After LPS binding to the pattern-recognition receptors, the increased expression of inflammatory cytokines, chemokines, and adhesion molecules direct the emigration of macrophages and neutrophils across the endothelial and epithelial barriers that separate the bloodstream from the pulmonary air spaces [8]. Tumor necrosis factor-*α* (TNF*α*) and interleukin-1*β* (IL-1*β*) are important mediators of lung inflammation during ALI [1]. In addition, the production of anti-inflammatory cytokine might limit the severity of the inflammatory response without interfering with the beneficial components of host defense and immunity [9–11].

Infiltrating leukocytes are hallmarks of lung inflammation associated with ALI. Macrophages are potent secretory cells that release proinflammatory cytokines, reactive oxygen species (ROS), and nitric oxide (NO), each of which has been implicated in the pathogenesis of lung injury [12]. Early response cytokines and mediators released by macrophages amplify inflammatory response by stimulating the nuclear factor-*κ*B- (NF-*κ*B-) dependent induction of proinflammatory mediators in cells [13]. Neutrophil response to inflammatory cytokine is also regulated by NF-*κ*B activation [11]. Macrophages and neutrophils release inflammatory mediators to defense harmful stimuli upon bacterial invasion; however, excessive inflammatory reaction leads to tissue damage and manifestation of pathological states. Therefore, targeting on uncontrolled inflammation seems feasible to control numerous inflammation-associated diseases [14].

 Sheng-Fei-Yu-Chuan-Tang (SFYCT), a formula designed on the basis of an empirical traditional Chinese medicine prescription composite of 13 medicinal plants (Table 1), has being used to treat acute or chronic lung diseases for decades in Taiwan. Our previous study has demonstrated the immunoregulatory effect of SFYCT on chronic allergic asthma using the *Dermatophagoides pteronyssinus* (*Der p*-) challenged chronic asthmatic murine model [15]. However, the immunoregulatory effects of SFYCT on acute lung inflammation have not been investigated. NF-*κ*B activation responding to LPS stimulation increases inflammatory cytokines, NO, and ROS production and amplify inflammatory responses during ALI. We hypothesized that SFYCT protects against LPS-induced ALI by modulating NF-*κ*B activity. Whether SFYCT possesses anti-inflammatory effects of decreasing inflammatory cytokines, NO, and ROS in LPS-stimulated macrophages and lung epithelial cells through inhibiting NF-*κ*B activity was investigated.

## 2. Methods

### 2.1. Mice and Reagents

Specific pathogen-free, male, and 6-week-old BALB/c mice from the National Laboratory Animal Center, Taiwan, were housed in microisolator cages and fed sterile food and water* ad libitum*. All experimental animal care and treatment followed the guidelines set up by the Institutional Animal Care and Use Committee of the China Medical University.

SFYCT (batch no. 98041021) was supplied by Koda Pharmaceuticals Ltd. (Taoyuan, Taiwan). The preparation was a mixture of 13 Chinese herbal medicines shown in Table 1. In brief, these were extracted with 1 L of boiled water twice for 1 hr. Poaching liquid was mixed two times. The dregs of the decoction were removed after filtering. The filtered liquid was lyophilized and crushed into a thin powder. The yield of the dried extract was about 38%. SFYCT was dissolved in distilled water and stored at −20°C before administration to mice.

### 2.2. Acute Lung Inflammation Murine Model

Male BALB/c mice, 6 weeks old, were anesthetized with a mixture of ketamine (80 mg/kg) and xylazine (30 mg/kg) given intraperitoneally. Intratracheal (I.T.) administration of LPS (*Escherichia coli*, 0055:B5; 100 *μ*g/kg) was performed with a bent 27G tuberculin syringe in a volume of 50 *μ*L. LPS-challenged mice were orally administered with 0.5, 1, or 2 mg/kg SFYCT (*n* = 6 in each group) 30 min before surgery. In addition, a group of 6 mice was not challenged (control group; *n* = 6). Twenty-four hr after LPS challenge, the mice were killed, and their lungs were harvested. Tissue specimens were immediately frozen in liquid nitrogen for RNA and protein extraction. Another group of mice underwent the same LPS I.T. challenging and SFYCT administration experiments, and their bronchoalveolar lavage fluid (BALF) was collected (two washes of 1 mL of ice-cold endotoxin-free PBS) according to a previously described procedure [16].

### 2.3. Detection of Nitrite (NO) Production

Production of NO was assessed as the accumulation of nitrite (NO_2_^−^) in the BALF of mice or medium using a colorimetric reaction with the Griess reagent. Briefly, samples were mixed with an equal (1 : 1) volume of Griess reagent (0.1% N-(1-naphthyl) ethylenediamine dihydrochloride, 1% sulfanilamide, and 2.5% H_3_PO_4_). The absorbance was measured at 540 nm using a 96-well microplate reader, and data were analyzed. Sodium nitrite was dissolved in double distilled water and then used as standards (from 1 to 50 mM).

### 2.4. Intracellular ROS Assay

Intracellular oxidative stress was measured by dichlorodihydrofluorescein diacetate oxidation. RAW264.7 cells (American Type Culture Collection, Rockville, USA) were plated at 1 × 10^5^/well in 96-well plates, cultured overnight, and washed twice with Hank's Buffered Salt Solution (HBSS) before experiments. Cells were exposed to 20 mM 5- (and 6-) chloromethyl-2′,7′-dichlorodihydrofluorescein diacetate, acetyl ester (CM-H_2_ DCFDA) (Invitrogen Life Technologies, Carlsbad, CA, USA) for 1 hr and then treated with HBSS containing the corresponding concentrations of LPS for 0.25 hr either with or without SFYCT 0.5 hr before treatment. Fluorescence was read immediately at wavelengths of 485 nm for excitation and 530 nm for emission on a fluorescence plate reader. The levels of ROS were calculated as a percentage increase compared with the control; the control was normalized to 100% of the basal level.

### 2.5. Measuring Cytokines

The levels of TNF*α*, IL-1*β*, IL-4, IL-6, and IL-10 of BALF were analyzed using ELISA kits (R & D Systems, Minneapolis, USA) as previously described [17]. Results are the means of duplicate assays.

### 2.6. Cell Culture and Reagents

The human lung epithelial cell line A549 (American Type Culture Collection, Rockville, USA) was maintained in 100 mm dishes with RPMI-1640 medium (Gibco BRL, Life Technologies, Inc., USA) supplemented with 10% (v/v) fetal bovine serum (FBS) at 37°C in a humidified atmosphere of 5% CO_2_. RAW264.7 murine macrophages were routinely grown on 100 mm dishes in Dulbecco's Modified Eagle's Medium (DMEM) with 2 mM L-glutamine and 15 mM HEPES supplemented with 10% FBS, 100 units of penicillin, and 100 mg/mL of streptomycin. Cultures were kept at 37°C in an atmosphere of 5% CO_2_. Cells were used at a passage of 7 to 10 in this study. 

### 2.7. MTT Assay

A549 cells were seeded in 96-well plates at a density of 10^4^ cells/well. After 12 hr, the cells were treated with LPS (100 ng/mL) for 24 hr combined with or without SFYCT treatment as indicated. The cells were then washed three times with PBS, and 500 *μ*g/mL MTT (3-(4,5-dimethylthiazol-2-yl)-2,5-diphenyltetrazolium bromide) solution (Sigma-Aldrich, USA) was added to each well; then the cells were incubated for 3 h. Formazan was made soluble by adding 200 *μ*L of dimethyl sulfoxide (DMSO) solution (Sigma-Aldrich, USA), and absorbance was measured at a wavelength of 570 nm using a microplate reader (Model 550; Bio-Rad Laboratories, Hercules, CA, USA).

### 2.8. Real-Time Reverse Transcriptase Quantitative Polymerase Chain Reaction (qPCR)

Total RNA of the lung tissue or RAW264.7 cells were extracted using a reagent (Trizol; Life Technologies, Rockville, MD, USA). Total RNA was subjected to reverse transcription using StrataScript H-reverse transcriptase to generate cDNA (Stratagene, La Jolla, CA, USA). To amplify the TNF-*α* and MCP-1 transcripts, real-time PCR was done using a kit (LightCycler FastStart DNA Master SYBR Green I kit; Roche Diagnostics, Indianapolis, IN, USA) according to the manufacturer's instructions. The gene-specific primer pairs (Table 2) were used in real-time qPCR. Individual PCR products were analyzed using melting-point analysis. Real-time qPCR product was analyzed using the comparative Ct method according to the manufacturer's instructions. Sample variation was corrected by subtracting the internal control gene, *β*-actin. 

### 2.9. Western Blotting

NF-*κ*B and iNOS in the lung tissue or RAW264.7 cells were detected by Western blot analysis. Protein extracts were prepared using the protein extraction kit (Panomics Inc., Santa Clara, USA). Extracts were separated by SDS-PAGE, transferred, and immobilized on a nitrocellulose membrane. The membrane was blocked by incubation with 5% nonfat dry milk in PBS for 2 hr and then hybridized with NF-*κ*B (1 : 500), phospho-NF-*κ*B (Ser536) antibody (1 : 1000), inducible NO synthase (iNOS), or *β*-actin (Cell Signaling, CA, USA) for 16 hr. Incubation with antibodies and detection of the antigen-antibody complex were performed using the ECL kit (Amersham Biosciences UK, Buckinghamshire, UK). Western blotting densities were analyzed using the Bio-Profil program and expressed as a fold increase relative to PBS controls. 

### 2.10. Statistical Analysis

The normality of data was analyzed by Kolmogorov-Smirnov test using SPSS 12.0 software. Data are presented as mean (SD). Multiple parametric comparisons were performed by one-way analysis of variance, followed by Dunnett's post hoc test. Statistical significance was set at *P* < 0.05.

## 3. Results

### 3.1. SFYCT Reduced Lung Inflammation and Attenuated Cytokine Production in LPS-Induced Acute Lung Injury Mice

**Figure 1:**
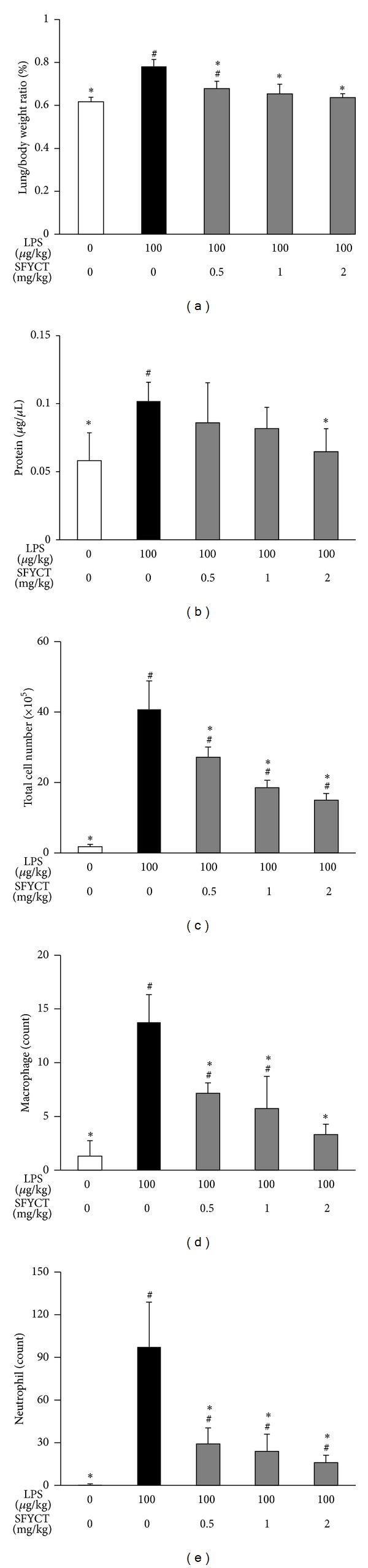
SFYCT reduced lung inflammation in LPS-induced ALI mice. BALB/c mice were intratracheally administrated with PBS, LPS, LPS + 0.5 mg/kg SFYCT, LPS + 1 mg/kg SFYCT, or LPS + 2 mg/kg SFYCT (*n* = 6 in each group). Twenty-four hours after the LPS challenge, lung/body weight ratio (a), protein concentration of BALF (b), total cell counts (c), macrophage counts (d), and neutrophil counts (e) of BALF were determined. Data are mean (SD). ^#^ *P* < 0.05  versus control group. *P < 0.05  versus LPS group.

Twenty-four hours after LPS administration, body weight of LPS group [18.2 (0.8) g] was lower than that of control group [20.1 (0.4) g]. One characteristic of ALI is the development of high-permeability edema accompanied with high protein content in the edema fluid. The lung/body weight ratio [0.78 (0.03)%] and protein concentration [0.10 (0.01) *μ*g/*μ*L] in the BALF were significantly higher in the LPS group than that in control group [0.61 (0.02)%; 0.05 (0.02) *μ*g/*μ*L]. SFYCT treatment dose dependently decreased the lung edema and protein content in the BALF after LPS stimulation (Figures 1(a) and 1(b)). A small amount of total cells, macrophages, and neutrophils were counted in the BALF of control mice. LPS intratracheal instillation elicited a massive recruitment of leukocytes [81.5  (16.1) × 10^5^], macrophages [13.7 (2.5) count], and neutrophils [97.3 (31.4) count] in the BLAF of mice in 24 hrs indicating the presence of inflammation during ALI. The series doses of SFYCT treatment significantly reduced the infiltration of leukocytes, including macrophages and neutrophils in the alveolar spaces (Figures 1(c)–1(e)).

**Figure 2:**
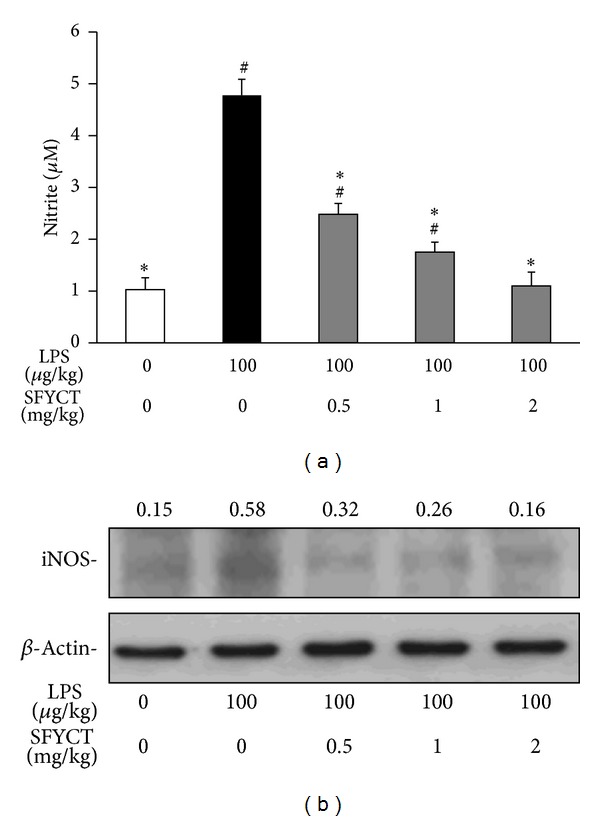
SFYCT reduced NO production and iNOS expression in LPS-challenged lung. (a) Nitrite concentration in the BALF of control and LPS-challenged mice with or without SFYCT (0.5, 1, and 2 mg/kg) (*n* = 6). Data are mean (SD). ^#^ *P* < 0.05  versus control group. *P < 0.05  versus LPS group. BALF was collected 24 h after the LPS challenge. (b) iNOS expression levels in the lung of mice were determined using Western blotting. The ratios of iNOS to *β*-actin are shown. *β*-actin was the internal control. Data are representative of three individual experiments.

Western blotting and the Griess reaction were used to determine the expression of iNOS and NO production, respectively. SFYCT treatment significantly decreased nitrite production in the BALF of mice after LPS stimulation (Figure 2(a)). SFYCT treatment also reduced LPS-upregulated iNOS expression (0.58 with LPS only versus 0.32, 0.26, and 0.16 with LPS + 0.5, 1, or 2 mg/kg SFYCT, Figures 2(a) and 2(b)) in the lung of mice after 24 h LPS treatment. 

**Figure 3:**
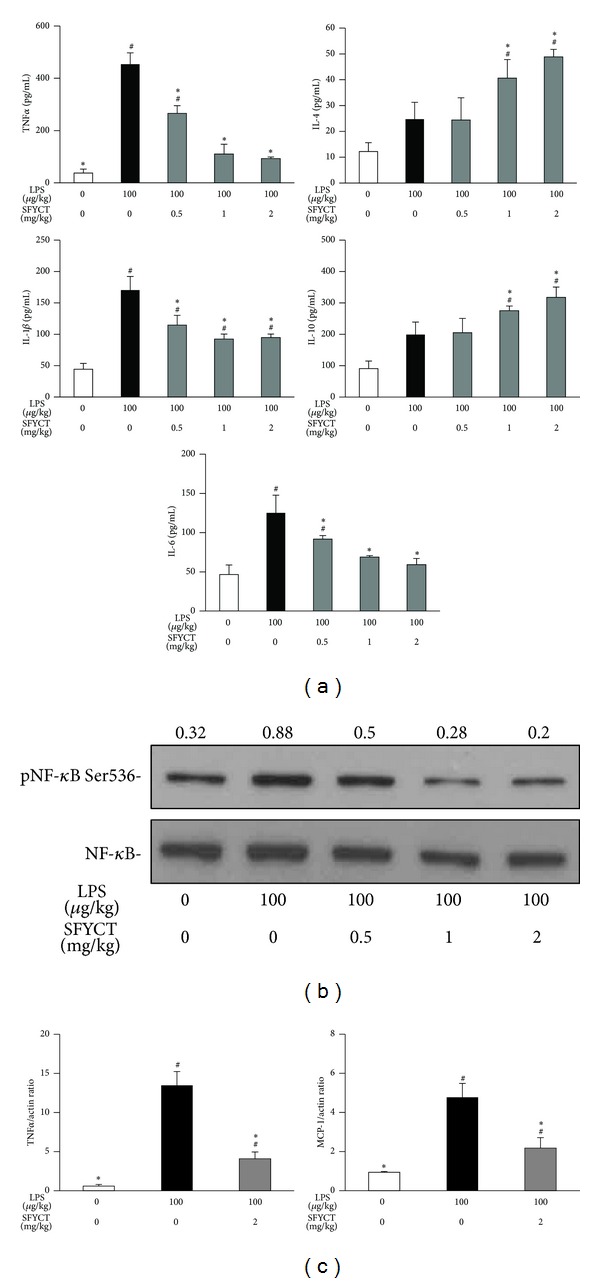
SFYCT reduced cytokine expression and NF-*κ*B phosphorylation in LPS-challenged mice. (a) Inflammatory cytokine (left panel), TNF*α*, IL-1*β*, and IL-6 concentrations, and anti-inflammatory cytokine (right panel), IL-4 and IL-10 concentration of BALF in normal or LPS-challenged mice combined with PBS or SFYCT treatment (*n* = 6 in each group). Data are mean (SD). ^#^ *P* < 0.05  versus control group. *P < 0.05  versus LPS group. BALF was collected 24 h after the LPS challenge. Lung was collected 24 h after the LPS challenge. (b) Phosphorylation of NF-*κ*B (Ser536) and iNOS expression were determined using Western blotting. The ratios of pNF-*κ*B to NF-*κ*B and iNOS to *β*-actin are shown, respectively. Data are representative of three individual experiments. (c) Expression of mRNA of TNF*α* and MCP-1 was analyzed using real-time RT-PCR (*n* = 6 in each group). Data are mean (SD). ^#^ *P* < 0.05  versus control group. *P < 0.05  versus LPS group.

We further examined the cytokine production in the BLAF of mice. LPS stimulation increased the concentrations of proinflammatory cytokines, including TNF*α*, IL-1*β*, and IL-6 [543.6 (87.1), 170.3 (43.6), and 125.2 (45.1) pg/mL, resp.] in the BALF of mice, but SFYCT treatment decreased those of cytokines (Figure 3(a), left panel). On the other hand, anti-inflammatory cytokines, IL-4, and IL-10 were secreted at basal level [12.2 (12.9) and 91.0 (47.8) pg/mL] in the BALF of control mice and elevated in that of LPS mice [24.7 (12.9), 199.2 (79.7) pg/mL]. The levels of these anti-inflammatory cytokines were significantly increased after LPS stimulation combined with SFYCT treatment. Utilizing Western blotting, we found that SFYCT treatment reduced LPS-induced phosphorylation of NF-*κ*B (Ser536) (0.88 with LPS versus 0.50, 0.28, 0.20 with LPS + SFYCT 0.5, 1.0, and 2.0 mg/kg, resp.) (Figure 3(b)). The mRNA expression of TNF*α* and MCP-1 in the lung was analyzed by real-time RT-PCR. LPS treatment significantly induced lung TNF*α* and MCP-1 mRNA [13.5 (1.7); 4.8 (0.7) ratio] expression levels. These inductions were decreased by SFYCT treatment (Figure 3(c)). 

### 3.2. SFYCT Reduced Cell Death and Decreased Cytokine Expression in LPS-Stimulated Lung Epithelial Cells In Vitro

**Figure 4:**
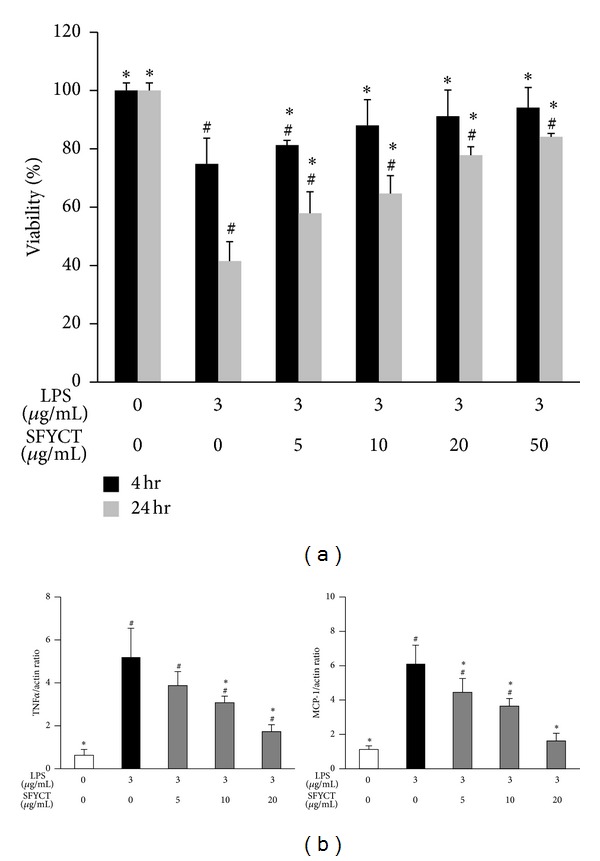
SFYCT reduced LPS-induced cytotoxicity and expression of TNF*α* and MCP-1 in lung epithelial cells. (a) A549 cells were treated with LPS (3 *μ*g/mL) with or without SFYCT at the indicated concentration for 4 and 24 hr. Cell viability in LPS-treated A549 cells was determined using MTT assay (*n* = 8). (b) Expression of mRNA of TNF*α* and MCP-1 was determined using real-time RT-PCR (*n* = 6). Data are mean (SD). ^#^ *P* < 0.05  versus control group. *P < 0.05  versus LPS group.

Four or twenty-four hours after LPS treatment, the cell viability of A549 cell was significantly lower than that of control cell [74.8 versus 100% at 4 hr; 41.5 versus 100% at 24 hour]. SFYCT treatment dose dependently increased the cell viability after LPS stimulation (Figure 4(a)). We also found that LPS increased the TNF*α* and MCP-1 mRNA expression of A549 cell [5.2 (1.3); 6.1 (1.1) ratio] while SFYCT treatment decreased the elevated cytokine expression (Figures 4(b) and 4(c)).

### 3.3. SFYCT Suppresses LPS-Induced Free Radical and Cytokine Production in RAW264.7 Murine Macrophages

**Figure 5:**
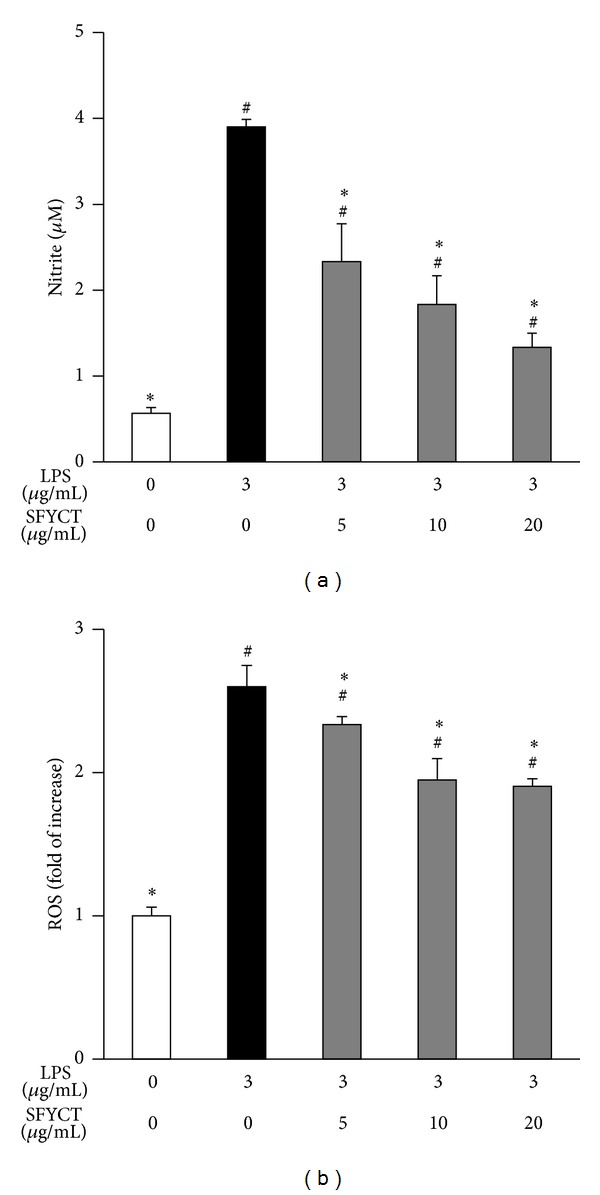
SFYCT decrease LPS-induced ROS and NO generation. RAW264.7 cells (5 × 10^4^ cells/well in 96-well culture plates; *n* = 8) were treated with LPS (3 *μ*g/mL) for 24 hr with or without SFYCT at indicated concentration for 0.5 hr. (a) Griess reagent was used to detect the generation of nitrite. (b) CM-H2 DCFDA was used to determine the generation of intracellular ROS. Data are mean (SD). ^#^ *P* < 0.05  versus control group. *P < 0.05  versus LPS group.

**Figure 6:**
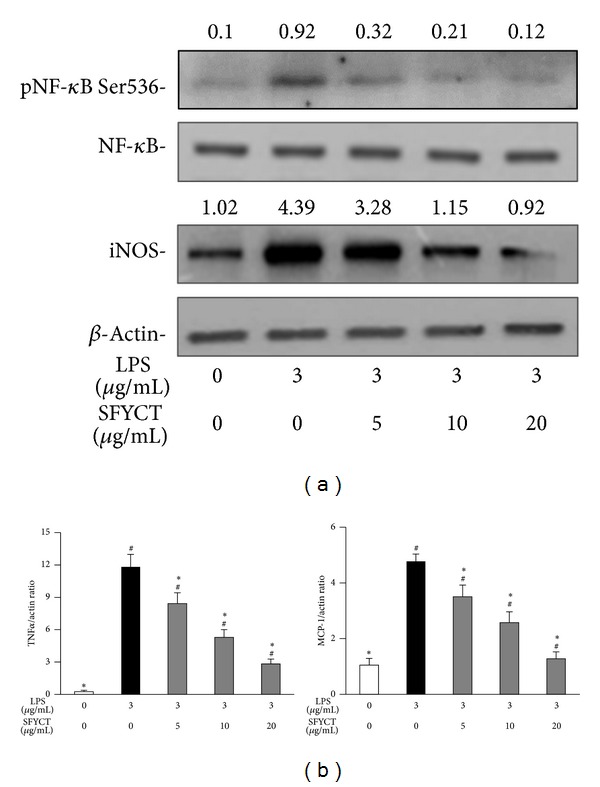
SFYCT reduced cytokine expression, NF-*κ*B phosphorylation, and iNOS expression in LPS-stimulated macrophage. RAW264.7 cells (1 × 10^6^ cells/well in 6-well culture plates) were treated with LPS (3 *μ*g/mL) for 2 hr with or without SFYCT at indicated concentration for 0.5 hr. (a) Phosphorylation of NF-*κ*B (Ser536) and iNOS expression were determined using Western blotting. The ratios of pNF-*κ*B to NF-*κ*B and iNOS to *β*-actin are shown, respectively. Data are representative of three individual experiments. (b) Expression of mRNA of TNF*α* and MCP-1 was analyzed using real-time RT-PCR (*n* = 6). Data are mean (SD). ^#^ *P* < 0.05  versus control group. *P < 0.05  versus LPS group.

To investigate whether SFYCT inhibits ALI by attenuating macrophage mediated immune responses, the RAW264.7 murine macrophage was stimulated with LPS with or without SFYCT treatment and analyzed. The results showed that LPS stimulation increased the NO [3.9 (0.01) *μ*M] and ROS [2.6 (0.15) fold] production in RAW264.7 murine macrophage. SFYCT treatment dose dependently decreased the LPS-induced NO and ROS generation (Figures 5(a) and 5(b)). SFYCT treatment reduced LPS-induced phosphorylation of NF-*κ*B (Ser536) (0.92 with LPS versus 0.32, 0.21, and 0.12 with LPS + SFYCT 5, 10, and 20 *μ*g/mL, resp.) in RAW264.7 cell 24 h after LPS stimulation. SFYCT treatment also reduced LPS-upregulated iNOS expression (4.39 with LPS only versus 3.28, 1.15, and 0.92 with LPS + SFYCT 5, 10, and 20 *μ*g/mL SFYCT, resp.) (Figure 6(a)) in RAW264.7 cell 24 h after LPS stimulation. We also found that LPS increased the TNF*α* and MCP-1 [11.8 (1.15); 4.8 (0.2) ratio] mRNA expression of RAW264.7 cell, but SFYCT treatment decreased the elevated cytokine expression (Figure 6(b)).

## 4. Discussion

ALI is characterized by accumulation of protein-rich edema fluid in alveolar space, overproduction of cytokines, and leukocyte recruitment [18, 19]. LPS-induced ALI model highlights way to explore mechanisms and discover new targets for drug development and therapy of human ALI [3–5]. This study discovered that SFYCT, a Chinese herbal medicine formula, effectively reduces LPS-stimulated proinflammatory cytokine but increases anti-inflammatory cytokine in LPS-induced ALI mice model. SFYCT also attenuates the pulmonary edema. We hypothesize that the mechanism whereby SFYCT protects LPS-induced ALI, at least in part, is via inhibition of NF-*κ*B phosphorylation.

Inflammation is an adaptive response triggered by infection, toxin, tissue injury, and irritation [20]. Upon exposure to bacterial infection, proinflammatory cytokines like TNF*α*, IL-1*β*, and IL-6 are secreted mostly by monocytes or macrophages to defend against bacterial infections [11]. Appropriate inflammatory response is beneficial for the host to protect against pathogens, but uncontrolled inflammation leads to tissue damage and manifestation of pathological states [21, 22]. Another subclass of cytokines is the anti-inflammatory cytokines, such as IL-4 and IL-10, which are involved in suppressing the activity of proinflammatory cytokines, hence downregulating the inflammatory response [23]. In present study, SFYCT treatment attenuated lung inflammation by inhibiting pro-inflammatory cytokine production but elevated anti-inflammatory cytokine production in the BALF of LPS-challenged mice (Figure 3(a)). Furthermore, IL-10 mediates anti-inflammatory effects by inhibiting the upstream NF-*κ*B transcription factor, an essential secondary messenger required for inducing proinflammatory cytokine gene expression [24]. We also observed that, with SFYCT administration, NF-*κ*B phosphorylation in the lung was inhibited, but IL-10 in the BALF was increased in LPS-challenged mice.

In the lung of LPS-induced ALI mice, monocytes are recruited to the alveolar space and differentiated into macrophages [25]. Macrophage and neutrophil not only clear pathogens but also produce cytokines and ROS, which profoundly affect endothelial, epithelial, and mesenchymal cells in the local microenvironment and contribute to host defense, tissue remodeling, and repair. MCP-1 is a chemoattractant for macrophage and neutrophil during inflammation [26–29]. We found that SFYCT significantly inhibited LPS-induced migration of neutrophils and macrophages to the bronchial space (Figure 1). This result seems related to SFYCT which decreased the mRNA expression of MCP-1 and TNF*α* in the lung of LPS-injected mice (Figure 3). We suggest that SFYCT could attenuate inflammation during acute lung injury by decreasing inflammatory mediators, MCP-1 and TNF*α*. Overproduction of ROS by activated macrophages is harmful to human ALI [12, 30–32]. LPS stimulation typically induces inducible nitric oxide synthase (iNOS)/nitric oxide (NO) biosynthesis [33]. Overproduction of NO by iNOS has been proposed to play a crucial role in the pathogenesis of inflammatory diseases [34, 35]. The antioxidative effect of SFYCT was demonstrated by its inhibitory effect on intracellular ROS production in LPS-stimulated macrophages and by reducing LPS-induced NO production and iNOS expression.

 NF-*κ*B is a major transcription factor regulating the expression of iNOS and inflammatory cytokines as well as mediating ROS production during LPS-induced inflammatory response [13, 36–38]. LPS-stimulated phosphorylation of I*κ*B leads to ubiquitination and degradation in macrophages. NF-*κ*B is free from complex and translocates to the nucleus, where it binds to DNA and induces activation of inflammatory response [13, 14]. We observed that phosphorylation of NF-*κ*B in the lung of mice or macrophages during LPS induced inflammatory responses. SFYCT treatment decreased the phosphorylation of NF-*κ*B while it attenuated LPS-induced lung inflammation. Taken together, we suggest that SFYCT could act as an effective anti-inflammatory medicine by inhibiting NF-*κ*B phosphorylation during ALI. The direct molecular evidence whether SFYCT interacts with transcription factors mediating immune responses would be clarified in future investigation.

There are currently no specific effective therapies for ALI; thus there is a great need for novel therapeutic approaches [4]. SFYCT has being prescribed to treat patients with acute and chronic lung diseases according to the theory of traditional Chinese medicine for decades in the Veterans General Hospital and Taichung Tsu Chi General Hospital, Taichung, Taiwan. So far, there is no side effect or toxicity argument from patients using SFYCT. Previously we had reported the possible immune regulatory effect on a *Dermatophagoides pteronyssinus* induced chronic asthmatic mice model [15]. This is the first study reporting that SFYCT has a marked anti-inflammatory effect in a clinically relevant model of ALI. Although LPS-induced injury model cannot exactly duplicate all features of human ALI since human ALI is seldom caused by any single event and is usually related to complicated interactions between primary and additional risk factors, variability and susceptibility to LPS may affect the relevance of animal data to the outcomes from human studies [39, 40]. LPS-induced injury is still a useful experimental in vivo model closely resembling ALI/ARDS in humans [4, 5]. In present study, we investigated one of the possible immune mechanisms whereby SFYCT prevents LPS induced ALI. Our animal experiments of LPS-induced ALI highlight the potential therapeutic strategy and provide support for using SFYCT when considering therapeutic approach. Whether the therapeutic effects of SFYCT on LPS-induced ALI could be reproduced in different ALI animal models or various risk factors induced ALI patients needs further investigation. Another limitation of present study result for clinical use is the prior administration of SFYCT before LPS-challenge which situation may not frequently occurs in clinical practice. It was reported that early application, 2 hours before LPS stimulation, of dexamethasone can reduce pulmonary inflammation and fibrosis after LPS-induced ALI in rats via the inhibition of TNF*α* mRNA expression [41, 42]. Several prior administration of antibiotics, such as florfenicol [43], tetracycline [44], and telithromycin [45], reduced the release of inflammatory mediators like TNF*α*, inhibited NF-*κ*B activation, and increased IL-10 production in LPS-induced ALI models. Here, we administrated SFYCT before LPS challenge and demonstrated that SFYCT showed its protective and immune regulatory effect in mice. These studies suggest that early administrating medicine could be considered to prevent ALI in critically injured patients at risk of developing ARDS.

In summary, Chinese herbal medicine formula, SFYCT, possesses anti-inflammatory effects in suppressing release of inflammatory cytokines but increasing anti-inflammatory cytokines production. SFYCT decreased inflammation-associated mediators including MCP-1, ROS, and NO while reduced iNOS overexpression inhibited cell migration to the alveolar space through suppressing LPS-induced NF-*κ*B activation in lung epithelial cells and macrophages. These results suggest that SFYCT protects against LPS-induced ALI in mice.

## Figures and Tables

**Table 1:** The ratio of the components in SFYCT.

Components	Amount (g)
Ginseng Radix (root of *Panax ginseng* C. A. Meyer)	4
Atractylodis Ovatae Rhizoma (root and rhizome of *Atractylodes macrocephala Koide*)	4
Citri Reticulatae Pericarpium (skin of fruit of *Citrus reticulate Blanco*)	4
Ephedrae Herba (stem of *Ephedra sinica* STAPF)	1.2
Mori Ramulus (branch of *Morus alba L.*)	4
Radix Bupleuri (root of *Bupleurum chinense DC*)	4
Cinnamomi Ramulus (root of *Cinnamomum cassia* BL)	4
Scutellariae Radix (root of *Scutellaria baicalensis* George)	4
Schizonepetae Herba (stem of *Schizonepeta tenuifolia* Briq.)	6
Sileris Radix (root of *Siler divaricatum* Benth et Hook f.)	6
Glycyrrhizae Radix (root of *Glycyrrhiza uralensis* Fisch)	4
Zingiberis Recens Rhizoma (root and rhizome of *Zingiber officinale* Rosc.)	2
Zizyphi Sativae Fructus (fruit of *Zizyphus jujube* Mill. Var. inermis Rehd.)	6

Total amounts	53.2

**Table 2:** Primer pairs used in this study.

Factor	Primer sequence (5′-3′)	
	Forward	Reverse
TNF*α*	GAGTGACAAGCCTGTAGCCCA	CCCTTCTCCAGCTGGAAGA
MCP-1	ACCTGCTGCTACTCATTCAC	TACAGAAGTGCTTGAGGTGG
*β*-Actin	GCTGGAAGGTGGACAGCGAG	TGGCATCGTGATGGACTCCG
